# *Cryptosporidium parvum*-induced neutrophil extracellular traps in neonatal calves is a stage-independent process

**DOI:** 10.3389/fvets.2023.1256726

**Published:** 2023-08-17

**Authors:** Magdalena Grabbe, Iván Conejeros, Zahady D. Velásquez, Seyed Sajjad Hasheminasab, Faustin Kamena, Axel Wehrend, Ulrich Gärtner, Anja Taubert, Carlos Rodrigo Hermosilla

**Affiliations:** ^1^Institute of Parasitology, Biomedical Center Seltersberg (BFS), Justus Liebig University Giessen, Giessen, Germany; ^2^Laboratory for Molecular Parasitology, Department of Microbiology and Parasitology, University of Buea, Buea, Cameroon; ^3^Clinic for Obstetrics, Gynaecology and Andrology of Large and Small Animals With Veterinary Ambulance, Faculty of Veterinary Medicine, Justus Liebig University Giessen, Giessen, Germany; ^4^Institute of Anatomy and Cell Biology, Justus Liebig University Giessen, Giessen, Germany

**Keywords:** calves, *Cryptosporidium parvum*, neonates, NETosis, polymorphonuclear neutrophils

## Abstract

**Introduction:**

Infections with the apicomplexan obligate intracellular parasite *Cryptosporidium parvum* lead to cryptosporidiosis—a worldwide zoonotic infection. *C. parvum* is one of the most common diarrheal pathogens in young calves, which are the main reservoir of the pathogen. Cryptosporidiosis leads to severe economic losses in the calf industry and being a major contributor to diarrhea morbidity and mortality in children. Polymorphonuclear neutrophils (PMN) are part of the innate immune system. Their effector mechanisms directed against invasive parasites include phagocytosis, production of antimicrobial molecules as well as the formation of so-called neutrophil extracellular traps (NETs). Like other leukocytes of the innate immune system, PMN are thus able to release chromatin fibers enriched with antimicrobial granular molecules extracellularly thereby immobilizing and partially killing invasive bacteria, viruses, fungi and parasites.

**Methods:**

*In vitro* interactions of neonatal bovine PMN and *C. parvum-oocysts* and sporozoites were illustrated microscopically via scanning electron microscopy- and live cell imaging 3D holotomographic microscopy analyses. *C. parvum*-triggered NETosis was quantified via extracellular DNA measurements as well as verified via detection of NET-typical molecules [histones, neutrophil elastase (NE)] through immunofluorescence microscopy analysis. To verify the role of ATP in neonatal-derived NETosis, inhibition experiments were performed with NF449 (purinergic receptor antagonist with high specificity to P2X1 receptor).

**Results and discussion:**

Using immunofluorescence- and SEM-based analyses, we demonstrate here for the first time that neonate bovine PMN are capable of forming NETs against *C. parvum*-sporozoites and oocysts, thus as a stage-independent cell death process. Our data further showed that *C. parvum* strongly induces suicidal neonatal NETosis in a P2X1-dependent manner, suggesting anti-cryptosporidial effects not only through firm sporozoite ensnarement and hampered sporozoite excystation, but also via direct exposure to NETs-associated toxic components.

## 1. Introduction

*Cryptosporidium parvum* is an apicomplexan protozoan zoonotic parasite of the Cryptosporiidae family within the phylum Alveolata. Recently, it has been reclassified as gregarine within the class Coccidea. *C. parvum* is the main causal agent of cryptosporidiosis, a waterborne and foodborne zoonotic disease resulting in severe enteritis with symptoms such as diarrhea, dehydration, abdominal pain, and weight loss. A global enteric multicenter study (GEMS) highlighted that *C. parvum* is the second leading cause, after rotavirus, of infant diarrhea, associated with toddler morbidity and mortality in developing countries (1). Neonatal calves are the main reservoir of *C. parvum* and thus have an important zoonotic potential in addition to the economic losses to the dairy and beef cattle industry. This is especially important in the first 6 weeks of life, although the clinical disease appears in the first 3 weeks after parturition. Infection of calves occurs by the uptake of sporulated oocysts that are shed in the feces of previously *C. parvum*-infected animals. Sporozoites are released in the upper intestine and infect the brush border of intestinal epithelial cells (IEC). They are localized intracellular but extracytoplasmic and forming parasitophorous vacuoles (PV) thereby causing atrophy of the microvilli and thus the corresponding symptoms. The infection with *C. parvum* is self-limiting in immunocompetent individuals because of the development of protective immune reactions that causes severe illness in immune-deficient hosts (2).

Early host innate immune reactions had proven to be important in the protective response to *C. parvum* infection in humans, mice, and cattle (3–7). Therefore, infiltration and attraction of neutrophils to the gut might play a crucial role in the elimination or reduction of *C. parvum* burden. In this context, PMN are the most abundant population in the bloodstream of cattle and had different mechanisms to control infections. These defense mechanisms include phagocytosis, degranulation, ROS production, and the formation of neutrophil extracellular traps (NETs) (8–10). To the authors' current best knowledge, this study represents the first report on neonate NET formation against *C. parvum*. Conversely, a few studies on NETs or METs against *C. parvum* or other parasites have exclusively used PMN, and monocytes obtained from adult individuals, which clearly differ from neonates in terms of the innate immune system (11–15). NETs are composed of chromatin extruded by activated PMN in the form of fibers. These fibers are decorated with enzymes and peptides that originated from PMN granules and thus have microbicide potential. These enzymes/peptides include but are not restricted to neutrophil elastase (NE), myeloperoxidase (MPO), lactoferrin, pentraxin, and global histones (H1, H2A/H2B, H3, and H4) (8). NET formation has been proven as an important defense mechanism against parasite species of the coccidian class such as *Toxoplasma gondii* (16, 17), *Besnoitia besnoiti* (13, 18–20), *Neospora caninum* (21) and also against large nematodes such as *Haemonchus contortus* (22), *Angiostrongylus vasorum* (14, 23), and *Dirofilaria immitis* (24). Recently, it is also described in the bovine system that the protozoan phylum euglenozoa *Trypanosoma brucei* induces strong different phenotypes of NETs, including aggregated NETs (aggNETs) (15). Previous studies have demonstrated that colostrum PMN derived from female dogs exhibit the ability to release NETs in response to viable *N. caninum* tachyzoites (25). Neonatal human neutrophils exhibited strong cellular aggregation and released NETs when exposed to fungal β-glucan and *Candida albicans* hyphae in the presence of extracellular matrix (26). Hence, neonatal neutrophils exhibit the ability to undergo NETosis in reaction to fungal stimulation. So far, adult human and bovine *C. parvum*-triggered NETs (11) have been reported and more importantly to be dependent on MPO, calcium influx, p38 MAPK signaling, and NE (7). In addition, *C. parvum*-induced METs in adult bovine monocyte have been reported and showed monocarboxylate transporter-dependent METosis (12). Released NETs not only prevented active host cell invasion of *C. parvum* sporozoites *in vitro* but also hampered sporozoite excystation from infective oocysts (11). In addition, bovine PMN exposed to *C. parvum* showed an increase in the expression of IL-6 and granulocyte-macrophage colony-stimulating factor (GM-CSF) (7). GM-CSF was recently reported as a modulator of several PMN responses such as MPO activity and NET formation in neonatal calves (27). It has long been known that there are significant differences between the immune systems of neonatal and adult mammals. In particular, in mammals with an epitheliochorealis placenta, such as bovine and other ruminants, there is only a small transfer of antibodies through the placenta during gravity from the maternal organism to the fetus. At birth, all essential immune components are present in the blood of the neonate, but it takes 2 to 4 weeks for them to reach full functionality and 5 to 8 months for complete immune system maturation in cattle. For this reason, the innate immune system of neonatal calves has a primary role in the first line of defense against invasive pathogens, including *C. parvum* (27).

Several receptors and signaling pathways have been involved in NET formation induced by parasites, such as monocarboxylate transporters (MCT) (28), toll-like receptors (TLR2 and TLR4) (29), and purinergic receptors (P2X and P2Y) (30). In the case of purinergic signaling, we have already demonstrated the pivotal role of the P2X1 ATP receptor in bovine neutrophil responses against *N. caninum* (21), *B. besnoiti* (20), and *T. brucei* (15). So far, the influence of inhibitors, targeting the P2X1 receptor, on neonatal bovine PMN confronted with *C. parvum* is unexplored.

## 2. Material and methods

### 2.1. Parasites and sporozoite excystation

*C. parvum* was maintained by serial passages of oocysts in 1-day-old calves at the Institute of Parasitology, University of Leipzig, Germany, as reported elsewhere (31). The *C. parvum* strain used here belongs to subtype 60-KDa glycoprotein (gp60) IIaA15G2RI, which is the most common zoonotic subtype to be reported in Germany and other industrialized countries (32–35). Oocysts were purified by a combined sedimentation/flotation technique according to the study mentioned in Najdrowski et al. (36), thereafter stored in sterile phosphate-buffered saline (PBS; pH 7.4), and supplemented with penicillin/streptomycin (200 μg/ml each; Sigma–Aldrich) and amphotericin B (5 μg/ml; Sigma–Aldrich) at 4°C until further use for a maximum of 6 months. The medium for *C. parvum* oocyst stock was changed at monthly intervals as previously reported (35).

For isolation of vital sporozoites, sporulated *C. parvum* oocysts (5 x 10^6^) were pelleted at 5,000 x *g* for 5 min at 4°C (35) before suspending them in an excystation medium. In brief, *C. parvum* sporozoite excystation was induced by supplementation of acidified (pH 2.0) and sterile pre-warmed (37°C) 1 x Hank's balanced salt solution (HBSS, Sigma–Aldrich) for 10–30 min at 37°C. Thereafter, free-released sporozoites were pelleted (5,000 x *g* for 5 min) and incubated in non-acidified 1 x HBSS for 10 min at 37°C. Following final centrifugation (5,000 x *g* for 5 min), sporozoites were re-suspended in sterile RPMI 1,640 cell culture medium (Gibco) without phenol red and supplemented with 0.3 g/l _L_-glutamine, 10% fetal bovine serum (FBS; both Gibco), 100 UI penicillin, and 0.1 mg streptomycin/ml (both Sigma–Aldrich). Only fresh and motile sporozoites were used for bovine PMN exposure and NET-related investigations.

### 2.2. Isolation of neonatal bovine PMN

Healthy neonatal calves (aged 2 to 6 weeks; *n* = 4) served as blood donors. In total, 10 ml of peripheral blood was collected by puncture of the jugular vein in 12 ml heparinized sterile plastic tubes (Kabe Labortechnik). The heparinized blood was diluted in 10 ml of sterile PBS with 0.02% ethylenediaminetetraacetic acid [(EDTA); Sigma–Aldrich], layered on 12 ml Histopaque 1,077 separating solution (density = 1.077 g/l, Sigma), and centrifuged (800 x *g*, 45 min, RT). After the removal of plasma, lymphocytes, and monocytes, the cell pellet containing erythrocytes and PMN was re-suspended in cold (4°C) sterile HBSS, treated with Red Blood Cell Lysis^®^ buffer (Sigma–Aldrich) for 10 min, and centrifuged (600 x *g*, 10 min, RT). Then, the pellet was washed in cold (4°C) HBSS, centrifuged (600 x *g*, 10 min, 4°C), and re-suspended in HBSS and left on the ice to rest (for 30 min) before use. Cells were counted in a Neubauer hemocytometer chamber. All blood samples were taken for the purpose of routine diagnostics in the Clinic for Obstetrics, Gynecology, and Andrology of Justus Liebig University Giessen (JLU), Giessen, Germany.

### 2.3. Flow cytometry F-actin polymerization assay

F-actin polymerization assay was performed using Alexa Fluor 488 phalloidin (Invitrogen), as described previously (37). In brief, 5 x 10^5^ neonatal PMN were co-incubated with *C. parvum* or plain medium for the negative controls for 3 h at 37°C and 5% CO_2_ in 12 x 75 mm polypropylene tubes (BD Falcon). Then, the cells were fixed with Fixation/Permeabilization^®^ solution (BD Biosciences, San Diego, CA, USA) and stored at 4°C until stained with Alexa Fluor 488 phalloidin (Invitrogen). First, an aliquot of 100 μl was centrifuged at 600 x *g* for 10 min at room temperature (RT). Then, the supernatant was discarded, and the sample was suspended in 100 μl of Alexa Fluor 488 phalloidin (1:1,000 in PBS with 1% BSA). After an incubation of 30 min at RT in the dark, the samples were centrifuged at 600 x *g* for 10 min, the supernatant discarded, and the samples were washed with 300 μl of sterile PBS before centrifuging once more at 600 x *g* for 10 min at RT. Finally, the cells were suspended in 400 μl of sterile PBS, and the acquisition was performed in the FL1 (FITC) channel in a BD Accuri C6 plus^®^ flow cytometer. Analysis was performed in FlowJo v 10.6.2 software over at least 10,000 events in the active gate.

### 2.4. Oxygen consumption rate (OCR) and extracellular acidification rate (ECAR) in *Cryptosporidium parvum-*exposed neonatal PMN

Activation of isolated bovine neonatal PMN was monitored using Seahorse XF^®^ analyzer (Agilent). In brief, 1 × 10^6^ PMN from three donors were pelleted at 500 × *g* for 10 min at RT. After removal of the supernatant, cells were re-suspended in 0.5 ml of XF^®^ assay medium (Agilent) and supplemented with 2 mM of _L_-glutamine, 1 mM pyruvate, and 10 mM glucose 1 × 10^5^ cells, corresponding to 50 μl of the cell solution. Cells were gently placed in each well of an 8-well XF^®^ analyzer plate (Agilent) and pre-coated for 30 min with 0.001% poly-_L_-lysine (Sigma–Aldrich). Then, 50 μl of XF^®^ assay medium (Agilent) was added to blank wells (=no cell controls). Finally, 130 μl of XF^®^ assay medium (Agilent) was added to all wells (180 μl total volume), and cells were incubated at 37°C without CO_2_ supplementation for 45 min before measurement by a Seahorse XF^®^ analyzer. Additionally, *C. parvum* oocysts were suspended in XF^®^ assay medium (Agilent; 300,000 oocysts/20 μl) and placed in one of the four injection ports of the instrument. For PMN controls, only 20 μl of XF^®^ assay medium (Agilent) was dispensed. The metabolic assay included basal measurement of three readings followed by injection of *C. parvum* oocysts (3 × 10^5^) or medium and 30 readings over time. The total assay duration was 240 min. Background subtraction, determination of OCR and ECAR, and the area under the curve (AUC) of obtained registries were calculated using Wave^®^ software (Desktop Version, Agilent), as reported elsewhere (14, 38).

### 2.5. Live cell imaging of neonatal bovine PMN confronted with *C. parvum* oocysts using 3D-holotomographic microscopy

Overall, 1 x 10^6^ isolated neonatal bovine PMN were centrifuged at 300 x *g* for 10 min at RT. The supernatant was carefully discarded, and cells were suspended in 2 ml of imaging medium containing 0.1% BSA (Sigma–Aldrich) and 2 μM DRAQ5 (Thermo Scientific). In total, 1 ml of this cell suspension was seeded in an Ibidi^®^ plastic cell plate with a 35 mm diameter and low profile. Then, the plate was placed in a top-stage incubation chamber (Ibidi) at 5% CO_2_ and 37°C. A resting time of 30 min was used to let neonatal PMN settle down in the plastic cell plate. Then, 2 x 10^6^ freshly isolated *C. parvum* oocysts were carefully added to the center of the plastic cell plate. Image acquisition was set for refractive index (RI; 3D tomography) and DRAQ5 channel (red) detection, applying time-lapse settings (image acquisition every 15 s over 120 min) using a Nanolive Fluo-3D Cell Explorer^®^ (Nanolive). At the end of the experiment, each channel was exported separately using Steve^®^ software v.1.6 (Nanolive) and managed with Image J^®^ software (Fiji version 1.7, NIH). In brief, the RI holotomographic reconstruction and the overlay of RI and DRAQ5 channels were obtained using the maximum intensity algorithm (Z project). For the digital staining based on RI values, the software Steve^®^ (Nanolive) was used, thereby applying the black body option with default settings.

### 2.6. Scanning electron microscopy

Neonatal bovine PMN (*n* = 4) were co-cultured with *C. parvum* oocysts and sporozoites (ratio 1:2) for 150 min on coverslips (10 mm of diameter; Thermo Fisher Scientific) and pre-coated with 0.01% poly-_L_-lysine (Sigma–Aldrich) at 37°C and 5% CO_2_. After incubation, cells were fixed in 2.5% glutaraldehyde (Merck), post-fixed in 1% osmium tetroxide (Merck), washed in distilled water, dehydrated, critical point dried by CO_2_ treatment, and sputtered with gold particles. Finally, all samples were visualized *via* a Philips XL30^®^ scanning electron microscope at the Institute of Anatomy and Cell Biology, JLU Giessen, Germany.

### 2.7. Visualization of neonatal NETs triggered by *C. parvum* via immunofluorescence microscopy analysis

Neonatal bovine PMN (*n* = 4) diluted in HBSS were seeded on 0.01% poly-_L_-lysine and pre-treated glass coverslips in a plastic 24-well plate (Greiner). PMN were stimulated with *C. parvum* oocysts (ratio 1:2), *C. parvum* sporozoites (ratio 1:2), Ca^2+^ ionophore (25 μM), and plain medium as negative control (37°C, 5% CO_2_). After 150 min of incubation, the samples were fixed with 2% paraformaldehyde for 15 min at RT, washed thrice with sterile PBS, and stored at 4°C until further use.

To visualize NET-specific structures/proteins, anti-histone (H1, H2A/H2B, H3, and H4, Merck #MAB3422) and anti-neutrophil elastase (Abcam #ab68672) antibodies were used. Therefore, the samples were blocked for 60 min at RT with PBS containing 2% bovine serum albumin (BSA; Sigma–Aldrich) and 0.3% Triton X-100 (Thermo Fischer Scientific). Then, the samples were incubated with the primary antibodies (1:200) for 180 min at RT in a wet chamber. Afterward, the samples were washed thrice in sterile PBS and incubated with the secondary antibodies (anti-mouse Alexa Fluor 594 A-11005; 1:500; and anti-rabbit Alexa Fluor 488 A-11008; 1:500) for 30 min at RT in the dark. Finally, the samples were washed three times with sterile PBS and mounted on Fluoromount-G^®^ with DAPI (Thermo Fisher Scientific) for 48 h at RT in the dark.

Visualization of neonatal *C. parvum*-triggered NETs formation was achieved using an inverted IX81^®^ epifluorescence microscope equipped with an XM 10^®^ digital camera (both Olympus) or was acquired with a ReScan Confocal Microscope^®^ instrumentation (RCM 1.1 Visible, Confocal.nl) equipped with a fixed 50 μm pinhole size and combined with a Nikon Ti2-A inverted microscope. The microscope was equipped with a motorized Z-stage (DI1500). The RCM unit was connected to the TOPTICA CLE laser with the following excitations: 405/488/561/640 nm. Images were taken *via* an sCMOS camera (PCO edge) using a CFI Plan Apochromat 60x lambda-immersion oil objective (NA 1.4/0.13; Nikon). The setup was operated by the microscope software NIS-Elements (version 5.11). Images were acquired *via* z-stack optical series with a step size of 0.1 microns depth to cover all structures of interest within the cells. Z-series were displayed as maximum z-projections. Identical brightness and contrast conditions were applied for each data set within one experiment using Image J software Fiji version (39).

### 2.8. Inhibition of purinergic receptor P2X1 of *C. parvum*-exposed neonatal bovine PMN

For P2X1-inhibition assays, freshly isolated neonatal bovine PMN were pre-treated with NF449 (10 μM, Tocris; purinergic receptor antagonist for P2X1) for 30 min and then co-cultured with *C. parvum* oocysts (1:2 PMN/oocyst ratio, 3 h, 37°C, 5% CO_2_). The inhibitor concentration was selected based on previous NET-related studies (15, 40).

### 2.9. Statistical analyses

For all experiments, in the current study, statistical significance was defined by a *p*-value of < 0.05, by applying non-parametric analyses: Mann–Whitney test when two experimental conditions were compared and Kruskal–Wallis test followed by Dunn's *post-hoc* test for multiple comparisons. All graphs (mean ± SD), AUC calculations, and statistical analyses were performed using GraphPad Prism^®^ software (v.7.03).

## 3. Results

### 3.1. *C. parvum* induces F-actin polymerization in neonatal bovine PMN

**Figure 1:**
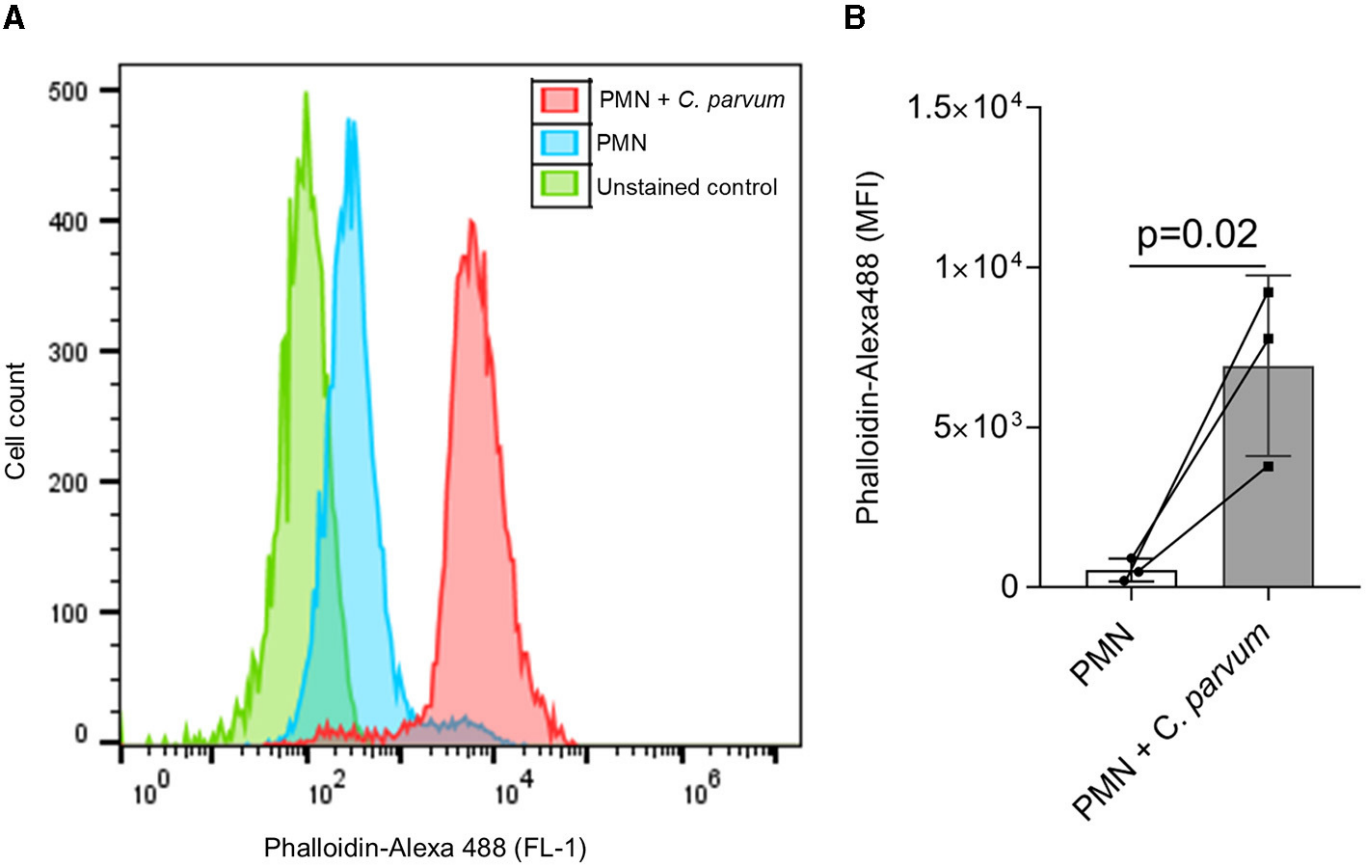
*C. parvum* oocysts induce neonatal PMN F-actin polymerization. In total, 1 x 10^5^ PMN were analyzed by flow cytometry using Alexa Fluor 488 phalloidin to detect F-actin. A shift to the right of the mean fluorescence intensity (MFI) is observed in the *C. parvum*-confronted condition **(A)**. The analysis of the fluorescence intensity as mean ± SD confirms this observation **(B)**. Statistical significance was defined as *p* < 0.05 applying a Mann–Whitney test (*n* = 3).

Initial activation of PMN after co-incubation with *C. parvum* was evidenced by the increase in F-actin polymerization and represented as a shift to the right in the FL1-emitted fluorescence (Figure 1A), and the mean ± SD of the detected fluorescence of Alexa Fluor 488 phalloidin in the *C. parvum*-confronted condition was increased compared with the unstimulated PMN (*p* = 0.02; Figure 1B).

### 3.2. Activation of neonatal bovine PMN by *C. parvum* analyzed by live cell 3D holotomographic microscopy

**Figure 2:**
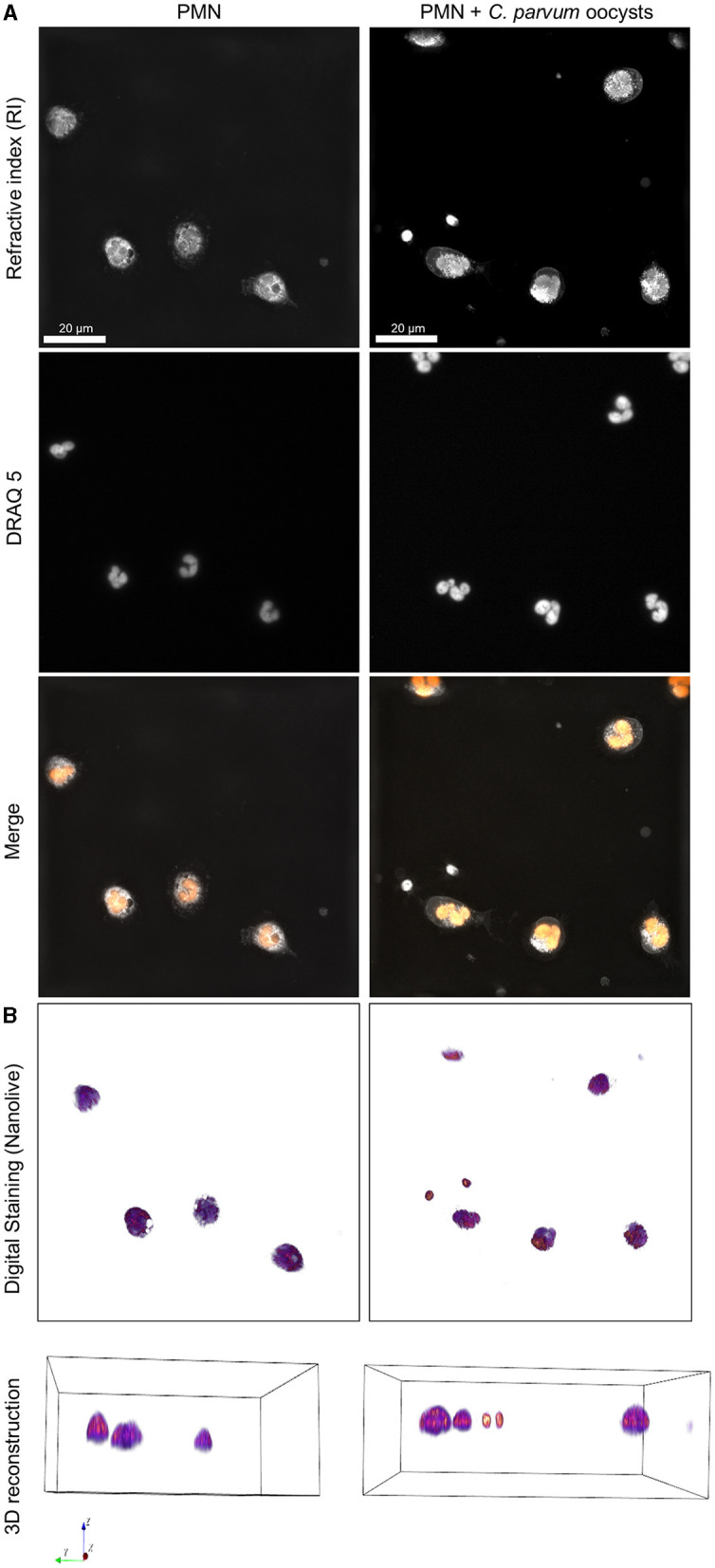
3D holotomographic microscopy of neonatal bovine PMN confronted with *C. parvum*. Overall, 1 × 10^6^ neonatal PMN were incubated in RPMI media containing 0.5% BSA and DRAQ5 2μM. Cells were registered using 3D holotomographic microscopy based on RI. **(A)** The nuclei stained by DRAQ5 and the merge of RI and DRAQ5 fluorescence channels are shown. **(B)** The digital staining based on the RI and 3D reconstruction is shown in the lower part.

The 3D holotomographic microscopy permitted us to observe in detail the initial reactions of neonatal PMN confronted with *C. parvum*. In addition, we determined that the diameter of non-stimulated neonatal bovine PMN is 8.93 ± 1.46 μm. The staining of the nuclear chromatin content with DRAQ 5 shows the typical multilobulated shape of the PMN nuclei (Figure 2). The digital staining and 3D reconstruction based on the RI also permit to identify granular structures on the cytoplasm.

The activation of neonatal PMN by *C. parvum* included the formation of pseudopods, typical crawling behaviors, and mobilization of cytoplasmic granules. Interestingly, no evident chromatin decondensation—a hallmark of NET formation—was observed (Supplementary Videos 5–9).

### 3.3. Average OCR and ECAR of neonatal PMN confronted with *C. parvum* oocysts

Current data show that no differences were observed in OCR and ECAR between non-stimulated neonatal PMN and *C. parvum*-confronted cells. The average values of OCR were 2.99 ± 0.38 O_2_ pmol/min for unstimulated neonatal PMN and 0.91 ± 0.44 O_2_ pmol/min for *C. parvum*-confronted PMN. In the case of ECAR, the average value for non-stimulated PMN was 5.23 ± 6.86 mpH/min, and for *C. parvum*-confronted PMN, the average value for non-stimulated PMN was 5.61 ± 6.66 mpH/min (Supplementary Figure 1).

### 3.4. *C. parvum* induces NETs in neonatal bovine PMN

**Figure 3:**
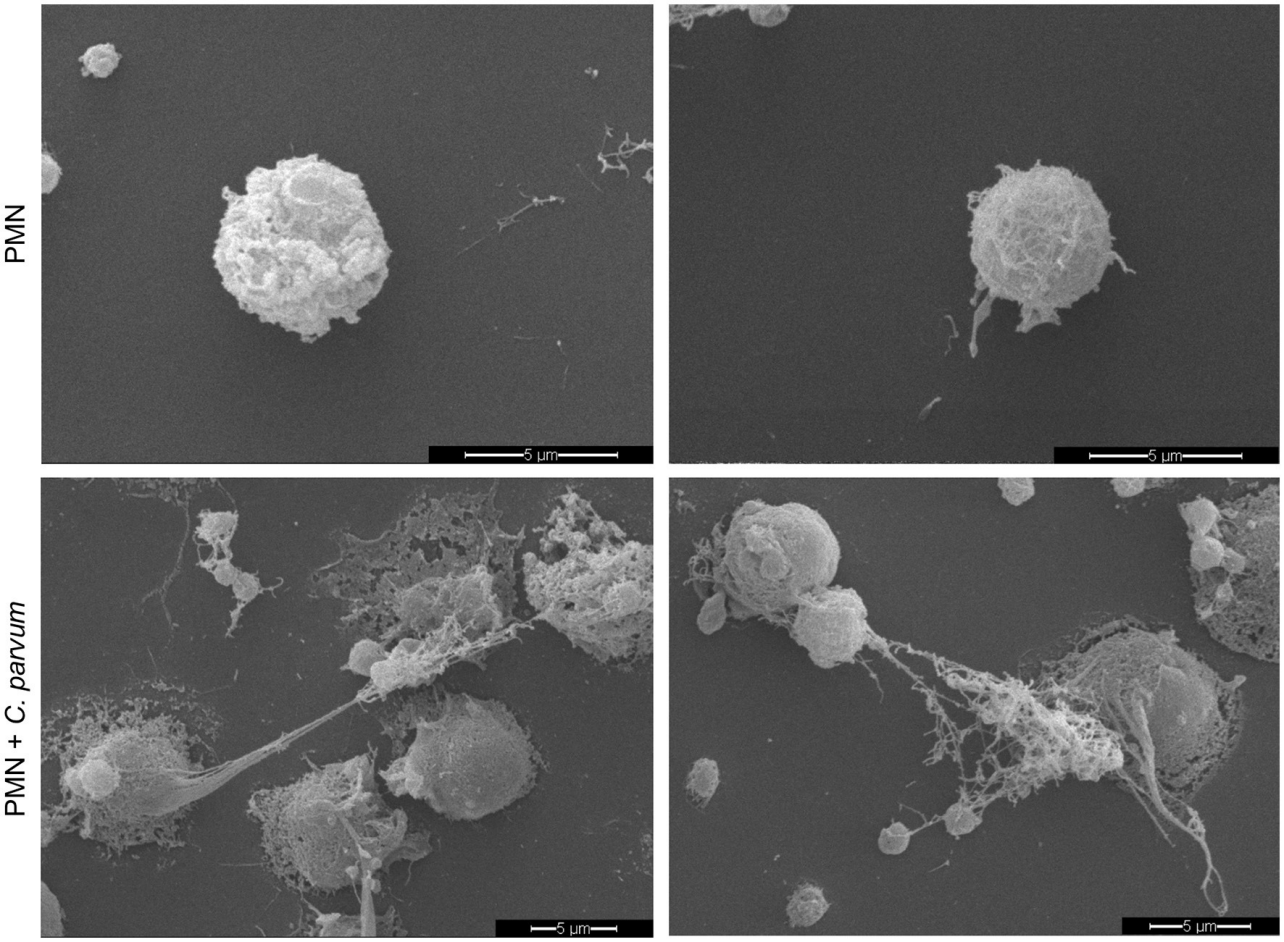
*C. parvum*-induced NETs in bovine neonatal PMN. Overall, 2 x 10^5^ non-stimulated neonatal PMN (upper panel) or *C. parvum* oocysts-confronted neonatal PMN (lower panel) were fixed after 180min, and the specimens were analyzed by scanning electron microscopy (SEM).

In total, 2 x 10^5^ non-stimulated neonatal PMN were confronted with *C. parvum* oocysts, and after 180 min of conformation, the samples were analyzed by SEM (Figure 3). Current data show that neonatal PMN are activated by the parasite confrontation. Compared with non-stimulated controls (Figure 3, Upper Panel), *C. parvum* induces membrane protuberances and formation of pseudopods and NET. NET formation was accompanied by the extrusion of a large amount of vesicles and granules, as depicted by SEM images.

### 3.5. *C. parvum* induces neonatal bovine NETs in a stage-independent manner

**Figure 4:**
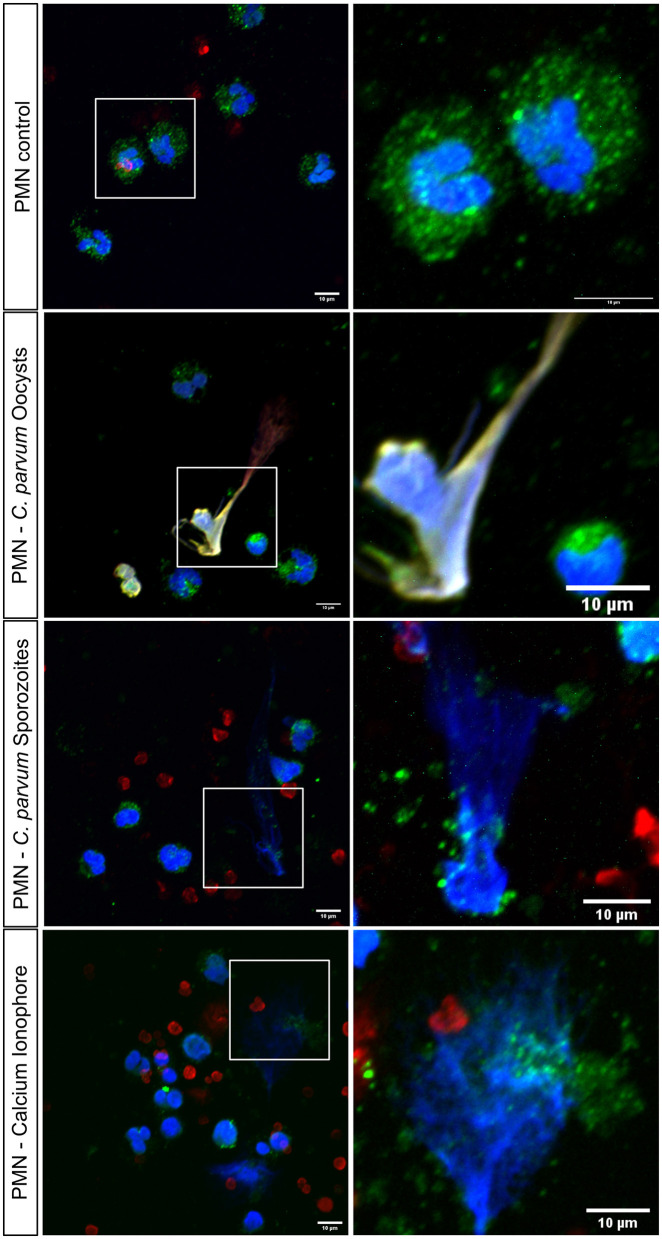
Stage-independent induction of bovine neonatal NETs by *C. parvum* oocysts and sporozoites analyzed *via* confocal microscopy. In total, 2 x 10^5^ neonatal bovine PMN were stimulated with oocysts and sporozoites of *C. parvum* for 180min and fixed. Shows NET formation by co-localization of DNA, blue, histones, red, and neutrophil elastase, green. Shows the positive control: neonatal bovine PMN with Ca^2+^ ionophore.

In total, 2 x 10^5^ neonatal PMN were confronted with *C. parvum* oocysts or sporozoites for 180 min, and subsequently, immunofluorescence for NET markers was performed. With DAPI, the DNA is shown in blue, a complex formed by histones H2A, H3, and H4 is shown in red, and neutrophil elastase (NE) is shown in green (Figure 4). Non-stimulated neonatal PMN served as control (Figure 4, Upper Panel), and neonatal PMN stimulated with the Ca^++^ ionophore A23187 served as positive control. Current data show that *C. parvum* oocysts and sporozoites induce neonatal NET formation, given the co-localization of extracellular DNA with NE. In the zoomed images (right panel), the granular distribution of NE on the extruded chromatin is observed. In addition, 3D reconstruction of unstimulated neonatal PMN and NETs induced by *C. parvum* oocysts, sporozoites, and the calcium ionophore A23187 is shown in Supplementary Videos 1–4, respectively.

### 3.6. P2X1-mediated ATP binding is of significant importance for neonatal NET formation against *C. parvum* oocysts

**Figure 5:**
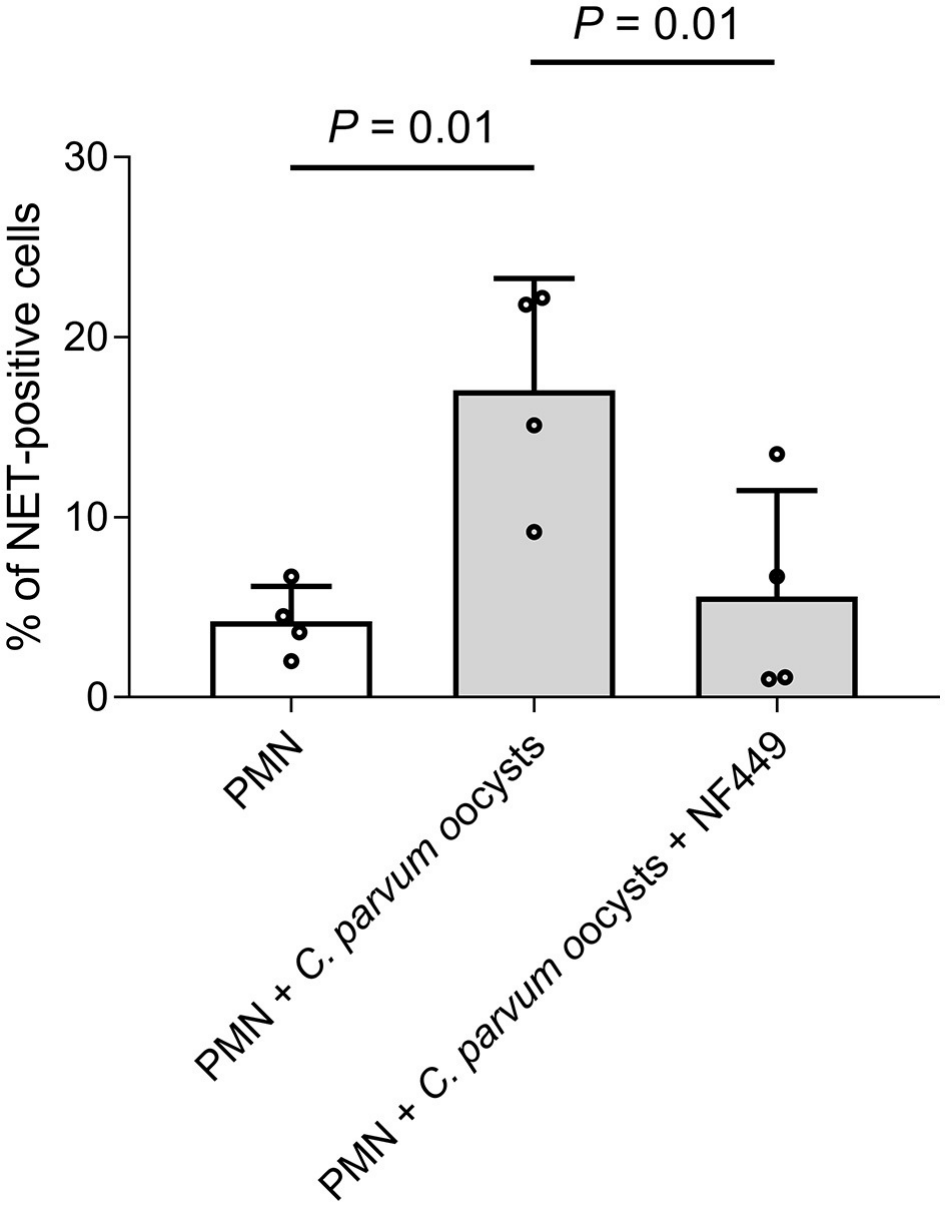
*C. parvum*-induced bovine neonatal NET is dependent on purinergic ATP binding. In total, 2 x 10^5^ neonatal bovine PMN were pre-treated with the inhibitor NF449 (10μM) for 30min, and *C. parvum* oocysts were added. Furthermore, there were two control groups: non-stimulated neonatal bovine PMN and neonatal bovine PMN, co-cultured with *C. parvum* oocysts. The DNA release was significantly inhibited when NF449 was used. *p*-values were calculated using ANOVA, followed by Dunnett's multiple comparison *post-hoc* test.

Overall, 2 x 10^5^ neonatal PMN were pre-treated with the purinergic receptor antagonist NF449 (10 μM) for 30 min and thereafter co-cultured with *C. parvum* oocysts for 180 min. Additionally, non-stimulated neonatal bovine PMN and neonatal bovine PMN co-cultured with *C. parvum* oocysts served as a comparison. Current data indicate that neonatal NET formation against *C. parvum* oocysts is an ATP-dependent process, while NET formation was significantly reduced when the purinergic receptor P2X1 was blocked (Figure 5).

## 4. Discussion

In this report, we focused on the neonatal bovine PMN response against different stages of *C. parvum*. In general, fetal innate immune responses are an understudied subject in mammals, although it is widely acknowledged that additional research is needed in the future. Of course, this affirmation is also true for the bovine system. Therefore, neonatal PMN have the ability to rapidly identify pathogen-associated molecular patterns (PAMPs), in order to initiate defense mechanisms. These PAMPs encompass various components found in microbial membranes, such as lipoproteins (known as TLR2 ligands), lipopolysaccharide (LPS; recognized as relevant TLR4 ligand), flagellin, bacterial nucleic acids, and molecules derived from fungi and parasites (30, 41). In this context, neonatal PMN have been reported as pivotal in response to pathogens such as *Staphylococcus aureus, E. coli*, and *Pseudomonas* species (42). This is the first systematic study of neonatal bovine PMN responses against different stages (i.e., oocysts and sporozoites) of zoonotic-relevant *C. parvum*.

At first, we evaluated the activation of *C. parvum*-confronted neonatal bovine PMN through F-actin polymerization as an indication of cytoskeleton changes that are involved in processes such as degranulation and NET formation (37, 43). Our results show that cytoskeleton remodeling is an important step in neonatal PMN activation, as it also occurs in adult-derived PMN (37, 44). The cytoskeleton rearrangement accompanied by movement of the granules, crawling, and pseudopod formation was evident when the *C. parvum*-confronted neonatal bovine PMN were analyzed by live cell 3D holotomographic microscopy. This technique permits detailed cell morphometric analysis and observation of cellular processes during the NETosis process, as reported elsewhere (45). Current data show that neonatal bovine PMN reacted to the addition of *C. parvum* oocysts and sporozoites within the first 10 min by increased pseudopod formation, chemotaxis, and movement of cytoplasmic granules and membrane protrusions. These results correlate with previous observations of adult PMN reacting against other apicomplexan parasites such as *B. besnoiti* bradyzoites (19). In addition, the morphometric analysis indicates that neonatal bovine PMN have an average diameter of 8.93 ± 1.46 μm and are smaller in size when compared with the reported average size of adult bovine PMN of 10–12 μm (46). Finally, the multilobulated nature of the PMN nuclei was also observed in both unstimulated and *C. parvum*-confronted neonatal PMN when the nuclear staining DRAQ5 was used. This is in line with several reports of neonatal PMN of humans and mice indicating no obvious differences in nuclear morphology between species or with adult bovine PMN.

NET formation induced by *C. parvum* was studied by SEM analysis and then confirmed by co-localization of DNA with typical NET markers including NE and histones. SEM permitted us to reveal ultrastructural phenotypical aspects of neonatal bovine PMN, highlighting the rugose nature of their cell membrane. In this context, in order to isolate neonatal PMN, we used different methodological approaches described to isolate PMN from adult bovine blood including the use of distilled water or phosphate-based buffer to lyse red blood cells (RBCs) (37, 47). Interestingly, the use of these two approaches showed poor performance for the isolation of neonatal bovine PMN. In contrast, the isolation yield was much better when we used a commercial RBC lysis buffer from the company Sigma–Aldrich, as described in the Material and methods section. We did not observe an evident pre-activated state of the cells by SEM, confocal microscopy, and/or live cell imaging by 3D microscopy analyses, suggesting that this phenotype is a characteristic of neonatal bovine PMN or indicative of increased adhesion and phagocytic activity, as previously suggested (48–50). *C. parvum* oocysts-triggered neonatal bovine NET formation was observed by SEM and thereafter confirmed by confocal microscopy. Here, the co-localization of extruded chromatin with NE and histones was demonstrated, and the 3D analysis of the fluorescence signal shows that neonatal bovine PMN are able to form spread NETs (*spr*NETs) upon stimulation not only with *C. parvum* oocysts but also with motile sporozoites. This observation is significant as both stages of the parasite have the potential to interact with neonatal polymorphonuclear leukocytes (PMN) *in vivo*. Our research group previously reported the NET formation in bovine PMN confronted with both, *C. parvum* sporozoites and oocysts (7, 11). In these former studies, NE and MPO were demonstrated on *C. parvum*-induced NET surfaces. In this study, the percentage of cells undergoing NETs was not reported, and thus, a direct comparison of the magnitude of NET formation observed in our study (i.e., 17% of neonatal were positive for NETs) is not possible. However, this value can be compared with NET formation induced by PMA in human neonatal PMN (15%) or stimulated with LPS (8%), indicating that neonatal PMN in general form less NETs than adult PMN (51, 52). The authors speculate that NET formation in neonatal PMN is dependent on the incubation time. Yost et al. (53) stimulated neonatal PMN for 1 h with LPS and PMA and did not find a significant amount of NET formation, whereas Lipp et al. (51) showed NET formation after 2 h with different stimuli. In addition, Marcos et al. (54) found NET formation in human neonatal PMN after 3 h, capable of killing bacteria. Byrd et al. (26) showed neonatal NET formation after 30 min of stimulation with fungal hyphae of *Candida albicans*, which suggests that neonatal NET formation might be dependent on time, stimulus, and also the age of the neonate. However, published data represent only human-derived PMN.

Purinergic signaling modulates several PMN functions, such as chemotaxis, ROS production, phagocytosis, degranulation, and importantly, NET formation against parasites (15, 30). In this study, we further confirmed the importance of the ATP purinergic P2X1 receptor since using the inhibitor NF449 reduces neonatal bovine NET formation induced by *C. parvum* oocysts. This finding is in line with a recent report on *C. parvum*-triggered NETosis in exposed adult bovine PMN, thereby suggesting purinergic signaling as pivotal in this ancient effector mechanism. Nonetheless, further detailed studies are necessary to better understand the molecular mechanisms behind different purinergic pathways, which might be involved in this well-conserved early innate defense response.

OCR can be used as a quantitative measure of PMN activation since one of the main responses of PMN is the oxidative burst that produces ROS (55). Interestingly, NET formation can be classified into ROS-dependent and ROS-independent (56). Current data show that *C. parvum* induces NETs in bovine neonatal PMN without an evident increase in OCR. However, we were able to report the basal OCR and ECAR values for neonatal bovine PMN for the first time.

Overall, we present the first characterization of neonatal bovine PMN reactions after exposure to *C. parvum* oocysts and sporozoites, spanning from phenotypical changes such as pseudopod formation, chemotaxis, and cell polarization to finally later responses such as NET formation. The role of P2X1 purinergic signaling in neonatal bovine NET formation must be further studied to clarify molecular aspects of this important defense mechanism not only in the bovine but also in the human immune system.

## Data availability statement

The raw data supporting the conclusions of this article will be made available by the authors, without undue reservation.

## Ethics statement

The animal study was approved by Justus Liebig University (JLU) Giessen Animal Care Committee Guidelines. Protocols were approved by the Ethic Commission for Experimental Animal Studies of the Federal State of Hesse (Regierungspräsidium Giessen; A9/2012; JLU-No.521_AZ), Germany, and in accordance to the European Animal Welfare Legislation: ART13TFEU and currently applicable German Animal Protection Laws. The study was conducted in accordance with the local legislation and institutional requirements.

## Author contributions

MG: Methodology, Writing–original draft. IC: Methodology, Writing–review and editing. ZV: Methodology, Writing–review and editing. SH: Methodology, Writing–original draft. FK: Conceptualization, Funding acquisition, Writing–review and editing. AW: Supervision, Writing–review and editing. UG: Supervision, Writing–review and editing. AT: Conceptualization, Funding acquisition, Supervision, Writing–review and editing. CRH: Conceptualization, Funding acquisition, Supervision, Writing–review and editing.
