# Supplementary figures and images for: Cryptosporidium parvum-induced neutrophil extracellular traps in neonatal calves is a stage-independent process
Source: Front Vet Sci. 2023 Aug 17;10:1256726. doi: 10.3389/fvets.2023.1256726 (PMC10470472; doi:10.3389/fvets.2023.1256726)

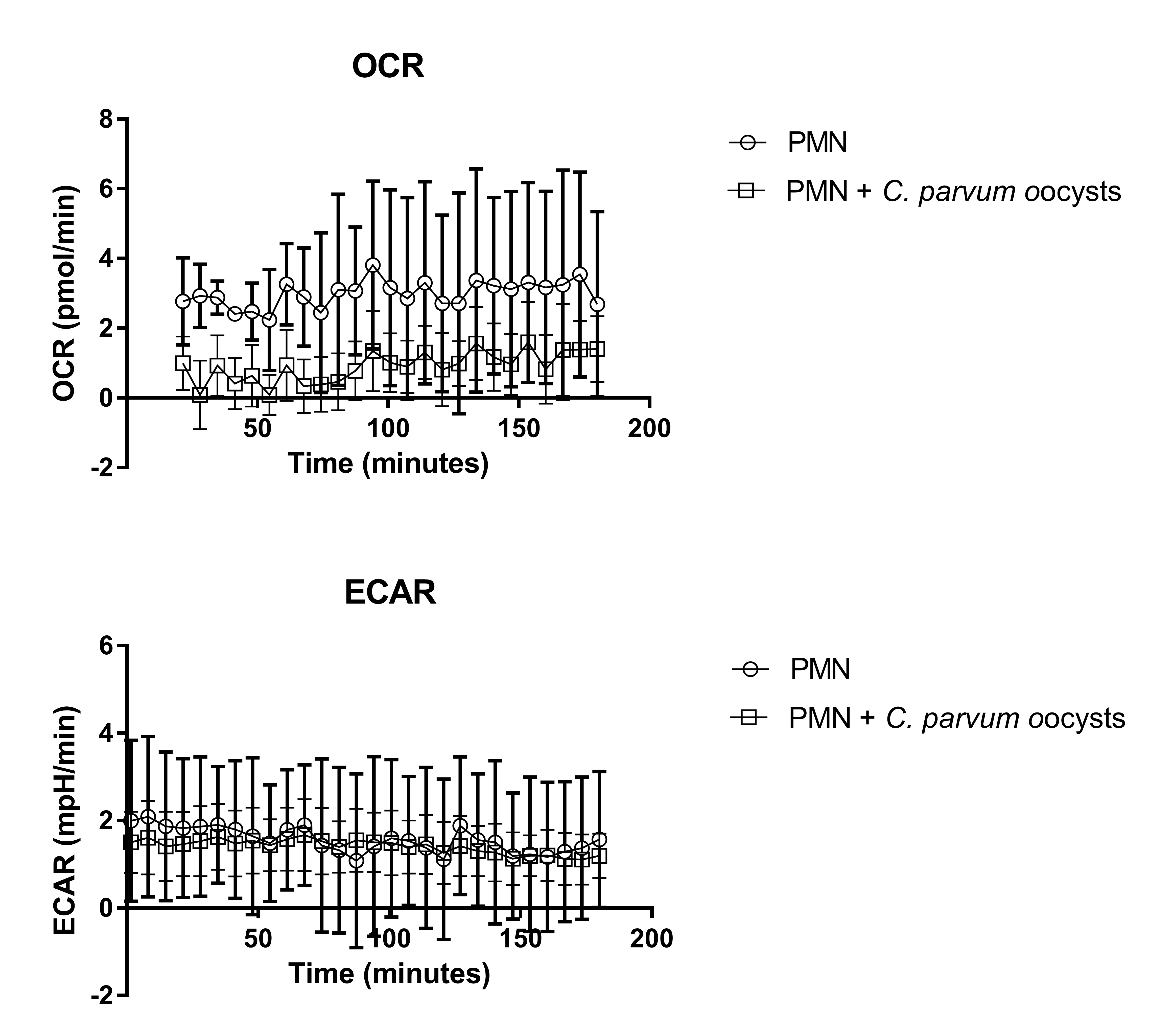

Supplement: Supplementary file 10 [file Image_1.TIF]
